# Exploring the Role of L10 Loop in New Delhi Metallo-β-lactamase (NDM-1): Kinetic and Dynamic Studies

**DOI:** 10.3390/molecules26185489

**Published:** 2021-09-09

**Authors:** Alessandra Piccirilli, Emanuele Criscuolo, Fabrizia Brisdelli, Paola Sandra Mercuri, Sabrina Cherubini, Maria Laura De Sciscio, Mauro Maccarrone, Moreno Galleni, Gianfranco Amicosante, Mariagrazia Perilli

**Affiliations:** 1 Department of Biotechnological and Applied Clinical Sciences, University of L’Aquila, 67100 L’Aquila, Italy; alessandra.piccirilli@univaq.it (A.P.); fabrizia.brisdelli@univaq.it (F.B.); sabrina.cherubini@graduate.univaq.it (S.C.); mauro.maccarrone@univaq.it (M.M.); gianfranco.amicosante@univaq.it (G.A.); 2 Department of Experimental Medicine, Tor Vergata University of Rome, Via Montpellier 1, 00121 Rome, Italy; emanuele.criscuolo@alumni.uniroma2.eu; 3 Macromolécules Biologiques, Centre d’Ingénierie des Protéines, InBioS, Université de Liège, 4000 Liège, Belgium; pmercuri@uliege.be (P.S.M.); mgalleni@uliege.be (M.G.); 4 Department of Medicine, Campus Bio-Medico University of Rome, Via Alvaro del Portillo 21, 00128 Rome, Italy; marialaura.desciscio@studio.unibo.it; 5 European Center for Brain Research/IRCCS Santa Lucia Foundation, Via Ardeatina, 306/354, 00179 Rome, Italy

**Keywords:** metallo-β-lactamases, NDM-1, kinetic studies, molecular dynamic

## Abstract

Four NDM-1 mutants (L218T, L221T, L269H and L221T/Y229W) were generated in order to investigate the role of leucines positioned in L10 loop. A detailed kinetic analysis stated that these amino acid substitutions modified the hydrolytic profile of NDM-1 against some β-lactams. Significant reduction of k_cat_ values of L218T and L221T for carbapenems, cefazolin, cefoxitin and cefepime was observed. The stability of the NDM-1 and its mutants was explored by thermofluor assay in real-time PCR. The determination of T_m_B and T_m_D demonstrated that NDM-1 and L218T were the most stable enzymes. Molecular dynamic studies were performed to justify the differences observed in the kinetic behavior of the mutants. In particular, L218T fluctuated more than NDM-1 in L10, whereas L221T would seem to cause a drift between residues 75 and 125. L221T/Y229W double mutant exhibited a decrease in the flexibility with respect to L221T, explaining enzyme activity improvement towards some β-lactams. Distances between Zn1-Zn2 and Zn1-OH- or Zn2-OH- remained unaffected in all systems analysed. Significant changes were found between Zn1/Zn2 and first sphere coordination residues.

## 1. Introduction

Antibiotic resistance in Gram-negative bacteria poses a serious threat to public health and β-lactamases are the main protagonist of this concern as a result of wide use, for about 70 years of β-lactam antibiotics [1]. Β-lactams are the most frequently used antibacterial agents, and, among them, carbapenems are those of choice to treat severe infection caused by Gram-negative pathogens [2]. The worldwide spread of carbapenem-inactivating enzymes (named carbapenemases) severely compromises the therapeutic efficacy of carbapenems. Carbapenemases are specifically included in Ambler Classes A, B and D [3,4]. Classes A and D enzymes are serine-β-lactamases whereas class B enzymes are metallo-β-lactamases (MBLs) which need zinc ions for hydrolysis activity. New Delhi metallo-β-lactamase-1 (NDM-1) is the last MBL to be discovered and it is widely spread all over the world. The *bla*_NDM-1_ gene is globally distributed in many bacterial genera with prevalence in *Enterobacterales*, specifically *K. pneumoniae*, *E. coli* and *E. cloacae* which cause a variety of hospital-acquired infections [5,6,7]. Moreover, the *bla*_NDM-1_ gene is frequently found in *A. baumannii* and *P. aeruginosa* [8,9,10,11]. Since its discovery, NDM-positive strains seem to be mainly widespread in the Indian subcontinent, yet they are associated with global travel and are found in many regions and countries [12]. NDM-1 is able to hydrolyse most β-lactams including carbapenems but not monobactams [13]. NDM-1, as well as the most clinically relevant MBLs (i.e., VIM and IMP-types), belongs to subclass B1 whose members require two zinc ions for catalytic activity [14,15]. NDM-1 is a monomeric enzyme which adopts αβ/βα fold and a metal binding motif which includes two zinc ions coordinated by six conserved residues. The Zn1 is coordinated by 3 histidine (3H site), including H120 (H116), H122 (H118) and H189 (H196), whereas Zn2 is coordinated by D124 (D120), C208 (C221) and H250 (H263) (DHC site) (BBL numbering is in parenthesis). For catalytic activity this enzyme needs one water molecule or in some cases hydroxide moiety (OH-) [16]. In MBLs the Zn ion at DHC site is essential in stabilising key reaction intermediates during substrate hydrolysis [17]. NDM-1 has five loops which play an important role in coordination of Zn ions, enzyme stability and substrate binding. In particular, L3 (roughly residues M67-G73) and L10 (roughly residues I210-A230) loops correctly orient β-lactams within the active site [18,19,20,21]. However, to make substrate hydrolysis possible, the active site needs both stability and flexibility and some residues of L6 and L10 loops seem to be involved [18]. Specifically, K125 (NDM numbering) and Y229 (NDM numbering) residues play an important role in stabilising L6 and L10 loops, respectively [18]. As described in several papers, amino acid substitutions in L3 and L10 loops seem to be associated with substrate hydrolysis [22,23,24].

Against this background, the aim of the present study was to investigate the role of residues L218 and L221 positioned in the L10 loop and residue L269 situated in helix α5 of NDM-1. Leucines 218, 221 and 269 (NDM numbering) form strong hydrophobic interaction with Y229 [18]. Leucines 218, 221 and 269 are not fully conserved in all subclass B1 MBLs. Indeed, position 218 is occupied by leucine (IMP, BlaB, BcII) or isoleucine (CcrA) or alanine (VIM); position 221 can be occupied by leucine (IMP) or valine (BcII, VIM) or isoleucine (CcrA, BlaB); position 269 can be occupied by histidine (VIM) or glycine (IMP). In this study, site-directed mutagenesis was performed to generate L218T, L221T, L269H and L221T/Y229W mutants. Threonine at position 218 and 221 was chosen because of its polar R groups whereas histidine 269 was chosen for similarity with VIM enzymes. The L218T was also evaluated in combination with Y229W substitution in the double mutant L221T/Y229W.

## 2. Results

### 2.1. Determination of k_cat_ and K_m_

Steady-state kinetic parameters of L218T, L221T, L269H and L221T/Y229W pure enzymes were determined against a large panel of β-lactams including carbapenems (imipenem and meropenem), penicillins (benzylpenicillin and carbenicillin) and 2nd, 3rd and 4th generation cephalosporins (cefazolin, cefoxitin, cefotaxime, ceftazidime and cefepime). Kinetic data of the four NDM-1 mutants (Table 1) were compared with those previously determined for NDM-1 and Y229W variants [25].

#### 2.1.1. Imipenem

Compared to NDM-1, L218T and L221T substantially decreased k_cat_ values 32- and 8-fold lower than NDM-1. Substitution Y229W, in the double mutant L221T/Y229W, was able to increase the k_cat_ values of L221T about 3-fold. With the exception of L218T, the NDM-1 mutants showed K_m_ values higher than NDM-1. Overall, NDM-1 mutants showed catalytic efficiency (k_cat_/K_m_) about 10-fold lower than NDM-1.

#### 2.1.2. Meropenem

L221T and L218T mutants showed k_cat_ values about 8- and 75-fold lower than NDM-1, along with a decrease, albeit minimal, of K_m_ values. The k_cat_/K_m_ value for L218T decreased 20-fold when compared with NDM-1. Both K_m_ and k_cat_ for L269H were slightly increased with respect to that determined for NDM-1 but catalytic efficiencies values were identical. The L221T/Y229W double mutant showed k_cat_ value 15-fold higher than that determined for L221T. Even so, the L221T and L221T/Y229W enzymes showed similar catalytic efficiencies.

#### 2.1.3. Benzylpenicillin

The k_cat_ values for L218T and L269H were about 3- and 5-fold higher than those of NDM-1. On the contrary, L221T showed lower k_cat_ value than NDM-1. Because of high K_m_ (937 μM) and low k_cat_ value, L221T enzyme showed a catalytic efficiency (k_cat_/K_m_) about 50-fold lower than NDM-1. L221T/Y229W hydrolysed benzylpenicillin very efficiently with k_cat_ value > 1000 s^−1^ but it showed higher K_m_ (>2000 μM). The k_cat_/K_m_ value was estimated to be 0.29 M^−1^s^−1^ (calculated using the same methods reported by Frère et al., 2020).

#### 2.1.4. Carbenicillin

The L218T, L221T and L269H enzymes showed K_m_ values comparable with that of NDM-1 whereas high K_m_ values were observed for L221T/Y229W and Y229W mutants. The k_cat_ values determined for L218T and L221T were half that of k_cat_ of NDM-1. L221T/Y229W showed a considerable increase of K_m_ and k_cat_ values with respect to NDM-1 and other mutants. The k_cat_/K_m_ value was estimated to be 0.29 M^−1^s^−1^ (calculated using the same methods reported by Frère et al., 2020). Substitution at position 269 (L269H mutant) did not significantly affect k_cat_ and K_m_ values which were slightly higher than those of NDM-1.

#### 2.1.5. Cefazolin

L218T, L221T and L221T/Y229W mutants showed lower k_cat_ values and, in addition, L218T and L221T exhibited k_cat_/K_m_ values approximately 10-fold lower than NDM-1. L269H and NDM-1 had the same k_cat_/K_m_ value but L269H exhibited double of k_cat_ and K_m_ values with respect to NDM-1. L221T/Y229W showed high K_m_ value and k_cat_ was quite similar to that of L221T; therefore, its k_cat_/K_m_ value was 5-fold lower than that of L221T.

#### 2.1.6. Cefoxitin

Compared to NDM-1, L218T and L221T mutants displayed very low k_cat_ values, resulting in lower k_cat_/K_m_ values. With respect to L221T, in the double mutant the hydrolytic activity towards cefoxitin was restored. However, high K_m_ value was determined (K_m_ > 5000 μM) (calculated using the same methods reported by Frère et al., 2020). As well as for benzylpenicillin and carbenicillin, the k_cat_/K_m_ value for cefoxitin was estimated to be 3.1 × 10^3^.

#### 2.1.7. Cefotaxime and Ceftazidime

The K_m_ values calculated for all mutants against cefotaxime and ceftazidime were quite similar to those of NDM-1, except for L221T/Y229W mutant which showed higher K_m_ value for ceftazidime. L221T decreased k_cat_ values 10-fold against cefotaxime but replacement Y229W restored enzyme activity towards this β-lactam. Concerning ceftazidime, L218T showed k_cat_ value higher than NDM-1 (45 s^−1^ vs. 18 s^−1^); instead, L221T and L269H showed k_cat_ value similar to NDM-1. L221T/Y229W showed k_cat_ value for ceftazidime lower than that of L221T.

#### 2.1.8. Cefepime

Cefepime was not a good substrate for all enzymes tested in this study. Indeed, k_cat_/K_m_ values of Y229W and L221T/Y229W were about 10-fold lower than NDM-1. In the case of cefepime, substitution Y to W at position 229 was unable to restore enzyme activity.

### 2.2. Effect of pH on K_m_ and k_cat_

Meropenem was chosen as substrate to study the effect of pH on K_m_ and k_cat_ values of NDM-1, L218T, L221T, L269H, Y229W and L221T/Y229W (Table 2). Our data showed that: (a) NDM-1 worked well at pH 7.0 (Hepes buffer); sure enough, both k_cat_ and k_cat_/K_m_ values were higher than those calculated at pH 6.5 (Bis-Tris buffer) and at pH 6.0 (MES buffer). K_m_ value of NDM-1 at pH 6.5 was half of that obtained at pH 6.0 and pH 7.0; (b) pH affected both K_m_ and k_cat_ values of L218T; indeed, k_cat_ value for meropenem increased 15- and 11-fold at pH 6.0 and 6.5, respectively and K_m_ values also increased at pH 6.0 and 6.5; (c) meropenem hydrolysis of L221T was not considerably affected by pH. Indeed, K_m_ value at pH 6.0, 6.5 and 7.0 was almost the same (ranging from 20 μM to 30 μM) but k_cat_ value at pH 7.0 was double with respect to that observed at pH 6.5 and 6.0; (d) k_cat_/K_m_ value of L269H at pH 6.5 was 2.5-fold higher than that determined at pH 7.0 and 6.0; (e) both L221T/Y229W and Y229W mutants worked better at pH 7.0. Specifically, k_cat_ at pH 7.0 of Y229W was 14-fold higher than that at pH 6.5 and pH 6.0.

### 2.3. Thermofluor Stability of NDM-1 and Its Mutants

To estimate the effect of the aforementioned amino acid substitutions in NDM-1, the stability of NDM-1, L218T, L221T, L269H, Y229W and L221T/Y229W was investigated by thermofluor assay in real-time PCR. The T_m_ values calculated as Boltzmann T_m_ (T_m_B) and derivative T_m_ (T_m_D) are shown in Table 3 and Figure S1. NDM-1 and its mutants showed different T_m_B and T_m_D values with a range of 0.28–7.79 °C and 1.32–9.22 °C, respectively. The NDM-1 and L218T had the highest melting temperature, indicating that they are the most stable enzymes.

### 2.4. Fluorescence Spectra

Fluorescence spectroscopy was used to analyse structural changes between NDM-1 and its mutants. Upon excitation at 280 nm, the intrinsic fluorescence of NDM-1 and its variants exhibited similar fluorescence emission maximum around 345 nm. NDM-1, Y229W and L221T/Y229W also showed the same fluorescence intensities, whereas a 52% decrease in L218T and L221T variants and a 46% increase in L269H mutant were observed with NDM-1 (Figure S2).

### 2.5. Molecular Dynamics Simulations

**Figure 1:**
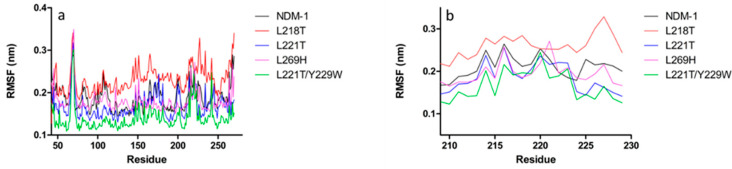
Comparison of Cα-atoms residual fluctuation of wild-type and mutant systems: (**a**) of all residues and (**b**) of just loop 10 residues (209–229).

In order to evaluate the role of substitutions for L218T, L221T, L269H and L221T/Y229W in NDM-1 (PDB code: 5ZGZ), a 20 ns of molecular dynamics simulations (MDs) was performed. Since protein stability is required for post-processing analysis, the root-mean-squared deviations (RMSD) of protein backbone atoms were monitored as a function of simulated time (Figure S3). All enzymes were quite stable. The root-mean-square fluctuations (RMSF) analysis highlighted two intense peaks in NDM-1 structure, characterising L3 (65–71) and L10 (209–229) residues (Figure 1a,b).

All mutants preserved this pattern of flexibility, showing an increased RMSF value in loop L3, while in loop L10 only L218T mutant fluctuates more than NDM-1 and other mutants. L221T mutant showed a similar trend to NDM-1, except for a drift between 75–125 residues; on the contrary, compared to L221T and NDM-1, the L221T/Y229W double mutant exhibited a systematic lower RMSF fluctuation. The L269H substitution led to an evident change in the fluctuation profile.

Since it is known that L10 loop is involved in the catalysis process, a specific RMSF backbone analysis of 209–229 residues was performed. As shown in Figure 1b, NDM-1 pattern of flexibility is marked by three peaks, matching K214, K216 and N220. This profile is conserved in both L221T and L221T/Y229W, with a decrease of intensity for the double mutant. In L221T and double mutant, residues positioned at C-terminal extremity of loop L10 are characterised by a loss in flexibility. Replacement L269H led to a decreased K214 flexibility and an enhanced fluctuation of L221. However, L218T mutant confirms the highest flexibility with an intense peak corresponding to E227 residue.

The average distances between Zn ions and atoms of the six residues of the “first sphere” of coordination (3H and DHC sites) were collected from the trajectory. As shown in Table 4, distances between ions (Zn1-Zn2, Zn1-OH-, Zn2-OH-) remain unaffected in all the systems analysed.

**Figure 2:**
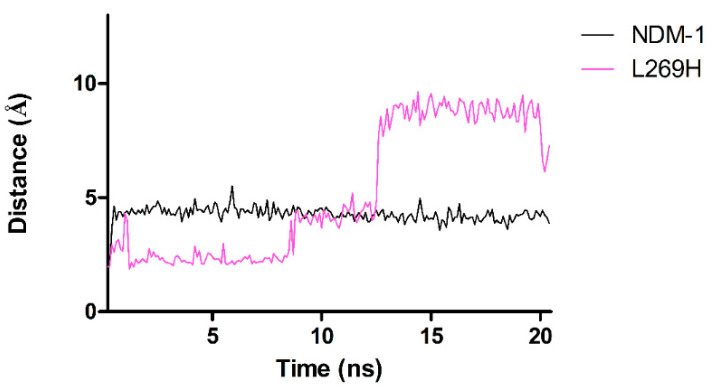
Evolution of the distances between Zn2 and H250 in NDM-1 and L269H mutant. Here, Zn2-H250 coordination is lost from 12.3 ns with a shift of about 3.5 A in comparison to NDM-1. Differences in the distances for Zn2-D124 were observed in L218T, L221T and L221T/Y229W enzymes. In these mutants, D124 was closer to Zn2 (Table 4).

The Zn1-Zn2 distance in L269H, L221T, L218T and L221T/Y229W was reduced to 2.9 Å, 2.18 Å, 1.89 Å and 1.83 Å, respectively. Significant change in coordination mode was observed in L269H mutant where H189 and H250 are further away from Zn1, whereas Zn1-H120 distance was reduced to 1.84 Å. In L269H mutant, the Zn2 in Zn2-H250 coordination was involved for approximately 60% of the simulation time; hence, for 40% of the time (from t = 12.3 ns), Zn2 lost the nitrogen coordination (Figure 2).

**Figure 3:**
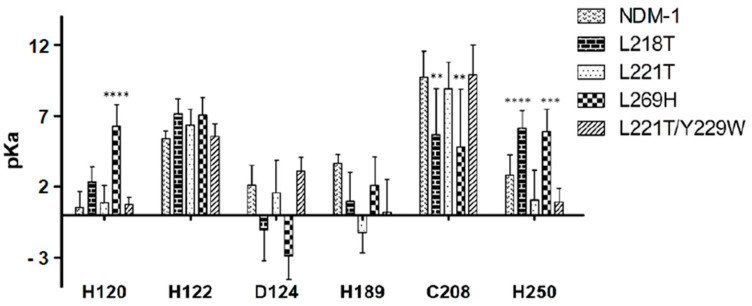
pKa values of the first sphere of coordination between NDM-1 and mutants calculated at pH 7. ** *p* = 0.003, *** *p* = 0.0003 and **** *p* < 0.0001, significantly different from NDM-1 by ANOVA test.

The pKa values, collected each 2 ns during the production run, were determined for all “first sphere” coordination residues in NDM-1 and its mutants (Figure 3). The L218T and L269H replacements in the NDM-1 structure caused a substantial alteration of pKa values in C208 and H250, in which a decrease of basic and acid behavior, respectively, was observed. In L269H mutant residue, H120 lost its strong acidic feature.

**Figure 4:**
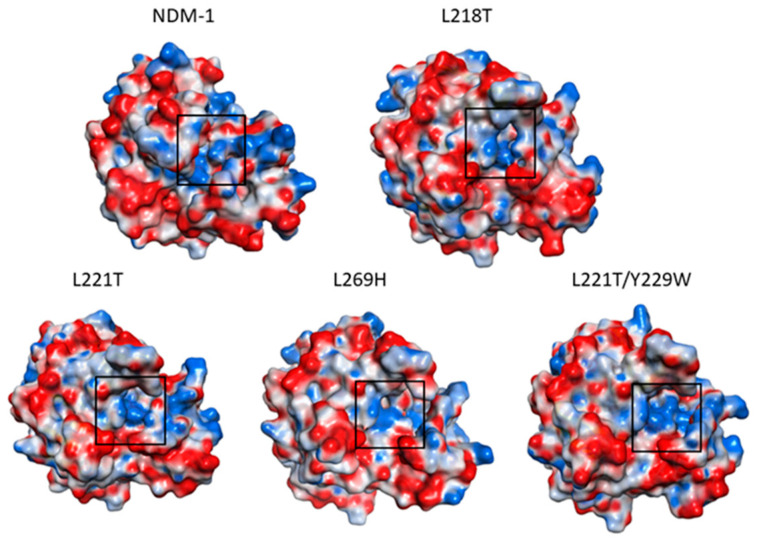
Electrostatic surface of all proteins: anionic surface in red, cationic surface in blue and neutral surface in white.

In addition, electrostatic surface of all systems, choosing the structures at 10 ns MD simulations was also reported (Figure 4). Focusing the attention on catalytic site of L218T, L221T and L269H mutants, a loss of cationic surface was noticed. Conversely, L221T/Y229W double mutant kept a very similar charge of NDM-1.

### 2.6. Molecular Dynamics Simulations for NDM-1 and L218T in Complex with Meropenem

**Figure 5:**
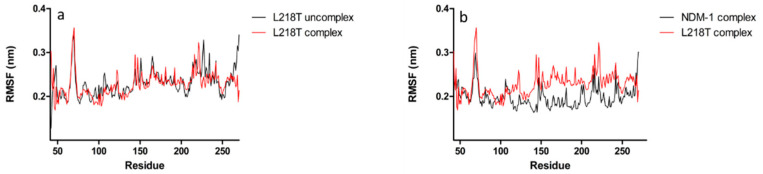
Evaluation of NDM-1 and L218T mutant flexibility related to meropenem interaction through comparison of root-mean-square fluctuations per residue. Flexibility pattern of (**a**) L218T mutant with (in red) and without (in black) meropenem, (**b**) NDM-1 (in black) and L218T mutant (in red) with meropenem.

As already seen in the analysis of the RMSF, L218T mutant showed a totally different profile from other systems; hence, a 20 ns molecular dynamics simulation with hydrolysed meropenem was launched. When monitoring RMSD (Figure S3) and RMSF, analysis of L218T mutant uncomplex and in complex with hydrolysed meropenem, an inversion of flexibility of E227 and T221 residues in loop L10 was observed (Figure 5a). Comparing L218T with NDM-1 in complex with hydrolysed meropenem, the L218T showed higher flexibility than NDM-1 (Figure 5b).

The average distances from the trajectory between Zn ions and atoms of the six residues of the “first sphere” of coordination (3H and DHC sites) in the NDM-1 and L218T complexed with hydrolysed meropenem were also collected and are reported in Table 5. The analysis revealed that in L218T/meropenem complex, the distances for Zn1-H122 and Zn1-Zn2 were much longer than the distance in NDM-1/meropenem complex.

## 3. Discussion

NDM-1 enzyme is a novel subclass B1 MBL of major clinical significance for its ability to hydrolyse a wide range of β-lactams. Since the first identification of NDM-1, many structures in native form and in complex with several β-lactams have been solved [16,18,19,26]. Here we interrogated the role of residues 218, 221 and 269 in substrate hydrolysis by replacing leucines at these positions with threonine (at 218 and 221) and histidine (at 269), respectively. Residues 218 and 221 are positioned inside the L10 which is involved in substrate hydrolysis by modulating the NDM-1 flexibility [18]. Our kinetic data stated that in both L218T and L221T, a significant reduction of k_cat_ values, with respect to NDM-1, was observed for imipenem, meropenem, cefazolin, cefoxitin and cefepime. NDM-1 has a tyrosine residue at 229 instead of tryptophan, as well as other subclass B1 MBLs, such as VIM-types, BcII and IMP-1. Substitution Y229W is present in a more active enzyme of subclass B2 (i.e., CphA) and B3 (i.e., L1) [22]. Chen et al. demonstrated that the Y229W substitution in NDM-1 improves the catalytic efficiency toward some β-lactams [21]. In our previous paper, we demonstrated that Y229W replacement was able to stabilise the L10 loop, restoring the hydrolytic activity of the mutant NDM-1^L209F^ which had completely lost activity towards a large panel of β-lactams [25,27]. In this study, the introduction, at position 229, of W in place of Y in the double mutant led to a notable increase of k_cat_ values towards benzylpenicillin and carbenicillin and a slight increase of k_cat_ values towards carbapenems, cefoxitin and cefotaxime. Different behaviour was observed for L269H mutant which, for the majority of β-lactams tested, showed an increase of k_cat_ and K_m_ values, but the resultant catalytic efficiency (i.e., k_cat_/K_m_) remained roughly constant.

Differences in melting temperature (T_m_) found between NDM-1 and its mutants would not seem to influence the enzyme activity. Indeed, the enzymes with lower T_m_ value were able to efficiently hydrolyse β-lactams. Similar behaviour has also been demonstrated in NDM-1 modified enzymes [24,27].

Fluorescence spectra are sensitive to the tryptophan environment because the fluorescence is strongly affected by the polarity of the surrounding area and can detect changes in protein conformation. The increase of fluorescence intensities at 345 nm in L218T and L221T and the decrease in L269H allowed us to estimate that substitutions in L10 loop and in helix α5 could change the microenvironment of aromatic fluorophores.

The different behaviour in kinetic profile observed in our NDM-1 mutants was explained by MD simulations. In fact, L218T fluctuated more than NDM-1 in L10. The L221T/Y229W double mutant exhibited a decrease in flexibility with respect to L221T, explaining enzyme activity improvement towards some β-lactams like meropenem, benzylpenicillin, carbenicillin and cefoxitin. Measuring the distance between Zn1 versus Zn2, Zn1 and Zn2 versus the six residues of the first sphere of zinc coordination in NDM-1 and its mutants, interesting considerations could be made. In NDM-1 and its mutants, the Zn1-Zn2 distance ranges from 3.18 to 3.28 Å. As regards the six residues of the first sphere of zinc coordination, the most important differences were retrieved for L269H mutant. It is noteworthy that H120, exhibiting neutral character, gets close to Zn1 (d = 1.84 Å) while H189, albeit retaining an acidic character, is further away (d = 4.27 Å), resulting in an inversion of coordination sphere compared to other mutants and NDM-1. In all NDM-1 crystallographic structures it has been observed that both zinc ions have the flexibility to move within the active site with a distance between Zn1 and Zn2 ranging from 3.5 Å without ligands to 4.6 Å with ligands [16,18,19]. In our study, the NDM-1 complexed with hydrolysed meropenem showed a Zn1-Zn2 distance of 4.54 Å in agreement with that found by Zhang et al. in NDM-1 in complex with hydrolysed ampicillin (Zn1-Zn2 distance of 4.6 Å) [28]. The same authors have stated that the shorter Zn1-Zn2 distance favours higher enzymatic activity [28]. In the present study, the L218T mutant in complex with hydrolysed meropenem showed a Zn1-Zn2 distance of 5.87 Å. This longer distance might correlate with lower enzymatic activity of L218T towards most β-lactams and, in particular, towards carbapenems. Correlation with active site conformational changes and enzymatic activity was also demonstrated in another subclass B1 metallo-β-lactamase, the BCII enzyme [29,30]

Replacement of leucines 218, 221 and 269 also affected the pKa of the six residues that coordinated Zn1 and Zn2. The drastic effect on pKa was observed for residues H120, C208 and H250. In particular, in L218T and L269H mutants, C208 showed a more acidic behaviour than NDM-1, while neutral character was recorded for H250. Alteration in pKa values affected protein electrostatic surface and, in fact, L218T and L269H lost their cationic surface with respect to NDM-1.

## 4. Conclusions

In summary, the replacement of leucine 218, 221 and 269 with threonine in NDM-1 affected (a) enzymatic activity, (b) the conformational fluctuations of L10 loop, (c) the distance between Zn1/Zn2 and “first sphere” residues, (d) the Zn1-Zn2 distance in L218T in complex with hydrolysed meropenem and e) the electrostatic surface of the enzymes. Data obtained in this study can open the way to design new molecules (substrates or inhibitors) that are more stable to the hydrolytic action of the metallo-β-lactamases. As a consequence, the discovery of these new substrates/inhibitors would lead to an improvement of therapy for severe infections caused by NDM metallo-β-lactamases-producing bacteria.

## 5. Materials and Methods

### 5.1. Site-Directed Mutagenesis

The plasmid pET-24/NDM-1 [31] was used as template to generate, by site-directed mutagenesis mutants, L218T, L221T and L269H using the overlap extension method as reported in our previous paper [32]. The double mutant L221T/Y229W was generated using pET-24-Y229W plasmid, as previously reported [25]. Each mutation was introduced into a PCR amplicon using mutagenic primers in combination with external primers NDM_for and NDM_rev (Table S1). Each PCR fragment, obtained from the overlap extension procedure, was sequenced using an ABI Prism3500 automated sequencer (Life Technologies, Monza, Italy).

### 5.2. Cloning of bla_L218T_, bla_L221T_, bla_L269H_ and bla_L221T/Y229W_

The mutated genes bla_L218T_, bla_L221T_, bla_L269H_ and bla_L221T/Y229W_ were cloned, without signal peptide, in pET-24a (+) vector using BamHI and XhoI restriction sites to obtain pET-24/L218T, pET-24/L221T, pET-24/L269H and pET-24/L221T-Y229W. Bacterial transformation was carried out, transferring recombinant plasmids into *E. coli* NovaBlue competent cells. The positive clones were selected on Luria-Bertani (LB) agar plates supplemented with kanamycin (50 mg/L). The authenticity of mutated genes was verified by sequencing both strands of the recombinant plasmids. For enzyme expression, the recombinant plasmids were transferred into *E. coli* BL21 (DE3)-Codon plus.

### 5.3. Production and Purification of L218T, L221T, L269H and L221T-Y229W Enzymes

*E. coli* BL21 (DE3)-CodonPlus cells harbouring pET-24/L218T, pET-24/L221T, pET-24/L269H and pET-24/L221T-Y229W were grown in 400 mL of tryptic soy broth (TSB) medium supplemented with kanamycin (50 mg/L) at 37 °C in an orbital shaker (180 rpm). Each culture was grown to achieve an absorbance value, measured at λ = 600 nm, of approximately 0.5 and, at this moment, 0.4 mM of isopropyl-β-d-thiogalactoside (IPTG) was added. Then, the cultures were incubated for 16 h at 22 °C under aerobic conditions. Crude enzymes were obtained by sonication in ice (5 cycles at 60 W with 2 min of break). The lysate was centrifuged at 22,000 rpm for 60 min, and the cleared supernatant was recovered and loaded onto a Q Sepharose Fast Flow column equilibrated with 25 mM Tris-HCl, pH 7.6. The metallo-β-lactamase was eluted with a linear gradient of 25 mM Tris-HCl (pH 7.6.) plus 1 M NaCl. The fractions containing β-lactamase activity were pooled, dialysed in 30 mM MES, pH 5.9, and loaded onto a Mono S column equilibrated with the same buffer, as reported in our previous paper [25]. The NDM-1 and Y229W enzymes were previously purified in our laboratories [25].

### 5.4. Determination of Kinetic Parameters

Steady-state kinetic parameters, K_m_ and k_cat_, were determined by measuring substrate hydrolysis under initial rate conditions and by using the Hanes linearisation of the Michaelis–Menten equation [33]. Reaction time-courses were monitored with the help of Perkin-Elmer Lamda 25 Spectrophotometer (Perkin-Elmer, Monza, Italy). The hydrolysis of substrate was measured, for all substrates, at 25 °C in Hepes 20 mM, pH = 7.0 + 20 μM ZnCl_2_. When the time-course was monitored with an initial substrate concentration S << K_m_, the slope of the line v_0_ versus [S] was considered to estimate k_cat_/K_m_ value if enzyme concentration (E_0_) is known [34]. The effect of pH was evaluated using three buffers, Hepes 20 mM, pH = 7.0 + 20 μM ZnCl_2_; Bis-Tris 20 mM, pH = 6.5 + 20 μM ZnCl_2_; MES 20 mM, pH = 6.0 + 20 μM ZnCl_2_, and measuring the initial rate of meropenem. Each kinetic value represents the mean of the results of three different measurements; the error rate was below 10%. The molar extinction coefficients and wavelengths used in the assay were imipenem Δε_M_^300^, −9000 M^−1^ cm^−1^; meropenem Δε_M_^29^7, −6500 M^−1^ cm^−1^; cefepime Δε_M_^260^, −10,000 M^−1^ cm^−1^; cefazolin Δε_M_^26^0, −7400 M^−1^ cm^−1^; cefoxitin Δε_M_^260^, −7775 M^−1^ cm^−1^; cefotaxime Δε_M_^260^, −7500 M^−1^ cm^−1^; ceftazidime Δε_M_^260^, −7500 M^−1^ cm^−1^; carbenicillin Δε_M_^235^, −780 M^−1^ cm^−1^; and benzylpenicillin Δε_M_^235^, −775 M^−1^ cm^−1^; nitrocefin Δε_M_^482^, −15,000 M^−1^ cm^−1^.

### 5.5. Fluorescence Assays

Fluorescence studies were carried out on a Perkin-Elmer LS-50B spectrofluorometer. The protein concentrations were 8 µg/mL. The buffer used was 20 mM Hepes buffer, 20 µM ZnCl_2_, pH 7.0. The excitation wavelength was 280 nm and the emission spectra, in the range of 300–500 nm, were recorded at 25 °C.

### 5.6. Thermofluor Assay

The thermal stability of NDM-1, L218T, L221T, L269H, Y229W and L218T/Y229W was determined using a fluorescence-based thermal stability assay (Protein Thermal Shift kit, Thermo Fisher Scientific, Monza, Italy) in a 7500 Fast Real-Time PCR System (Applied Biosystem, Monza Italy). The protein melt reaction mix (20 μL total) was added to the wells of the 96-well PCR plate. The melt reaction mix included 1 μg of each enzyme, protein shift dye (8×) and 20 mM Hepes pH 7 + 20 μM ZnCl^2^. The plate was heated from 25 °C (2 min) to 99 °C (2 min) with heating rate of 1 °C/min. The fluorescence intensity was measured with Ex/Em:490/530 nm. Analysis of Boltzmann T_m_ (T_m_B) and derivative T_m_ (T_m_D) was carried out by Protein Thermal Shift Software Version 1.4. The melting temperature T_m_B was calculated by fitting data in the region of analysis to the Boltzmann equation (Protein Thermal Shift Software, Thermo Fisher Scientific, Monza, Italy) whereas T_m_D was calculated for the region of analysis using the derivative of the melt curve (Protein Thermal Shift Software).

### 5.7. Molecular Dynamics Simulations

Molecular dynamics simulations (MDs) were carried out using MOE software 2020.09 (Chemical Computing Group, Montreal, QC, Canada) [35]. The crystal structure of NDM-1, without co-crystallised molecules (PDB code: 5ZGZ) was retrieved from Protein Data Bank with a resolution of 0.95 Å [28,36]. Mutants were built through Protein Builder MOE tool. Before MD analysis, selected enzymes were protonated using standard MOE protocol. Then, a cubic box, enabling the periodic system, was built and filled with water molecules. The entire system, using 0.1 RMS k_cal_/mol/Å2 gradient with AMBER10:EHT force field was minimised. A time step of 1.0 fs was employed after an initial energy minimisation of 100 ps; the system was gradually heated from 10 to 330 K in 100 ps. The simulations were performed in the NVT ensemble (T = 300 K) for 100 ps and NPT ensemble (T = 300 K) at a constant pressure of 1 bar for 200 ps. The production run for MD simulations was carried out for 20 ns for both free enzymes and enzyme-ligand complex. The latter was obtained from the selected enzyme and the hydrolysed meropenem (retrieved from 5YPM structure) [37]. In this latter case, the whole system was prepared using the same aforementioned procedure. The Cα-RMSD and Cα-RMSF as post-processing analyses were evaluated and distances from trajectory, using the equilibrated structure as reference, were collected. The pKa values of coordinating residues were monitored during the simulations (at pH 7) and they were picked each 2 ns during the production run and were reported as average.

## Figures and Tables

**Table 1:** Kinetic constants of L218T, L221T, L269H and L221T/Y229W compared with NDM-1 and Y229W.

Substrates	Variant	K_m_ (μM)	k_cat_ (s^−1^)	k_cat_/K_m_ (M^−1^ s^−1^)
Imipenem	NDM-1 *	35 ± 1	64 ± 3	1.8 × 10^6^
	L218T	13 ± 1	2 ± 0.1	1.5 × 10^5^
	L221T	59 ± 4	8 ± 1	1.3 × 10^5^
	L269H	149 ± 9	40 ± 3	2.7 × 10^5^
	L221T/Y229W	129 ± 6	29 ± 1	2.2 × 10^5^
	Y229W *	81 ± 3	38 ± 1	4.7 × 10^5^
Meropenem	NDM-1 *	80 ± 2	75 ± 2	9.4 × 10^5^
	L218T	22 ± 2	1 ± 0.1	4.5 × 10^4^
	L221T	20 ± 1	9 ± 1	4.5 × 10^5^
	L269H	117 ± 7	109 ± 8	9.3 × 10^5^
	L221T/Y229W	174 ± 6	135 ± 3	7.7 × 10^5^
	Y229W *	259 ± 18	820 ± 5	3.2 × 10^6^
Benzylpenicillin	NDM-1 *	250 ± 10	105 ± 5	4.2 × 10^5^
	L218T	832 ± 20	294 ± 2	3.5 × 10^5^
	L221T	937 ± 25	78 ± 5	8.3 × 10^4^
	L269H	541 ± 12	549 ± 5	1.0 × 10^6^
	L221T/Y229W	>2000	>1000	0.29
	Y229W *	841 ± 35	552 ± 3	6.6 × 10^5^
Carbenicillin	NDM-1 *	285 ± 5	108 ± 4	3.8 × 10^5^
	L218T	265 ± 10	59 ± 3	2.2 × 10^5^
	L221T	398 ± 15	48 ± 3	1.2 × 10^5^
	L269H	339 ± 9	347 ± 5	1.0 × 10^6^
	L221T/Y229W	>2000	>1000	0.29
	Y229W *	1176 ± 25	866 ± 6	7.3 × 10^5^
Cefazolin	NDM-1 *	20 ± 1	42 ± 1	2.1 × 10^6^
	L218T	74 ± 5	9 ± 1	1.2 × 10^5^
	L221T	25 ± 3	8 ± 1	3.2 × 10^5^
	L269H	41 ± 2	83 ± 2	2.0 × 10^6^
	L221T/Y229W	9 ± 1	14 ± 1	1.5 × 10^6^
	Y229W*	48 ± 2	191 ± 4	4.0 × 10^6^
Cefoxitin	NDM-1 *	26 ± 1	23 ± 1	8.8 × 10^5^
	L218T	31 ± 3	3 ± 0.2	9.6 × 10^4^
	L221T	31 ± 3	1.5 ± 0.1	4.8 × 10^4^
	L269H	100 ± 5	11 ± 1	1.1 × 10^5^
	L221T/Y229W	>5000	>11	3.1 × 10^3^
	Y229W *	2100 ± 25	4 ± 0.2	2.0 × 10^3^
Cefotaxime	NDM-1 *	14 ± 1	20 ± 1	1.4 × 10^6^
	L218T	24 ± 2	30 ± 2	1.2 × 10^6^
	L221T	10 ± 1	2 ± 0.1	2.0 × 10
	L269H	37 ± 2	37 ± 1	1.0 × 10^6^
	L221T/Y229W	10 ± 1	14 ± 1	1.4 × 10^6^
	Y229W *	52 ± 4	107 ± 3	2.1 × 10^6^
Ceftazidime	NDM-1 *	50 ± 4	18 ± 0.5	3.7 × 10^5^
	L218T	19 ± 1	45 ± 3	2.4 × 10^6^
	L221T	32 ± 1	19 ± 1	5.9 × 10^5^
	L269H	81 ± 4	10 ± 1	1.2 × 10^5^
	L221T/Y229W	222 ± 10	7 ± 1	3.0 × 10^4^
	Y229W *	250 ± 15	10 ± 1	4.0 × 10^4^
Cefepime	NDM-1 *	35 ± 5	13 ± 1	3.7 × 10^5^
	L218T	39 ± 2	3 ± 0.1	7.7 × 10^4^
	L221T	40 ± 3	2 ± 0.2	5.0 × 10^4^
	L269H	64 ± 3	11 ± 1	1.7 × 10^5^
	L221T/Y229W	152 ± 8	3 ± 0.5	2.0 × 10^4^
	Y229W *	117 ± 8	2.5 ± 0.1	2.0 × 10^4^

Each kinetic value represents the mean of the results of three different measurements; the error rate was below 10%. * Data were from reference [25].

**Table 2:** Effect of pH on K_m_, k_cat_ and k_cat_/K_m_ of meropenem for L218T, L221T, L269H, Y229W and L221T/Y229W.

Meropenem	HEPES 20 mM 20 μM ZnCl_2_ pH 7.00			BIS TRIS 20 mM 20 μM ZnCl_2_ pH 6.5			MES 20 mM 20 μM ZnCl_2_ pH 6.0		
	K_m_ (μM)	k_cat_ (s^−1^)	k_cat_/K_m_ (μM^−1^ s^−1^)	K_m_ (μM)	k_cat_ (s^−1^)	k_cat_/K_m_ (μM^−1^ s^−1^)	K_m_ (μM)	k_cat_ (s^−1^)	k_cat_/K_m_ (μM^−1^ s^−1^)
NDM-1	80 ± 2	75	0.94	46 ± 2	18	0.39	98 ± 3	40	0.41
L218T	22 ± 2	1	0.04	166 ± 7	11	0.06	189 ± 8	15	0.08
L221T	20 ± 1	9	0.45	30 ± 2	5	0.16	28 ± 2	4	0.14
L269H	117 ± 7	109	0.93	42 ± 3	96	2.28	182 ± 7	167	0.92
L221T/Y229W	174 ± 6	135	0.77	298 ± 18	67	0.22	101 ± 4	36	0.36
Y229W	259 ± 18	820	3.17	72 ± 4	67	0.93	104 ± 5	60	0.57

Each kinetic value represents the mean of the results of three different measurements; the error rate was below 10%.

**Table 3:** Effect of replacements L218T, L221T, L269H, Y229W and L221T/Y229W on thermal stability (T_m_) of NDM-1.

Enzymes	T_m_B (°C)	ΔT_m_B (°C)	T_m_D (°C)	ΔT_m_D (°C)
NDM-1	50.66	−	53.73	−
L218T	50.38	−0.28	52.41	1.32
L221T	49.06	−1.60	46.55	−7.18
L269H	42.87	−7.79	44.51	−9.22
Y229W	46.62	−4.04	47.86	−5.87
L221T/Y229W	45.02	−5.64	46.36	−7.37

**Table 4:** Average distances in Å between Zn and their coordinating atoms, collected from the trajectory.

	NDM-1	L218T	L221T	L269H	L221T/Y229W
Zn1-Zn2	3.26 ± 0.05	3.28 ± 0.05	3.27 ± 0.05	3.18 ± 0.10	3.27 ± 0.06
Zn1-OH-	1.70 ± 0.03	1.71 ± 0.03	1.70 ± 0.03	1.68 ± 0.03	1.71 ± 0.03
Zn2-OH-	1.66 ± 0.02	1.68 ± 0.03	1.68 ± 0.03	1.69 ± 0.03	1.66 ± 0.02
Zn1-H120	4.30 ± 0.49	4.13 ± 0.28	4.29 ± 0.29	1.84 ± 0.04	4.18 ± 0.26
Zn1-H122	4.98 ± 0.84	4.40 ± 0.46	4.59 ± 0.47	4.37 ± 0.95	4.64 ± 0.52
Zn1-H189	1.96 ± 0.07	1.93 ± 0.06	1.96 ± 0.07	4.27 ± 0.66	1.94 ± 0.07
Zn2-D124	3.27 ± 0.51	1.89 ± 0.10	2.18 ± 0.63	2.90 ± 0.99	1.83 ± 0.30
Zn2-C208	1.95 ± 0.03	2.00 ± 0.05	1.99 ± 0.05	1.98 ± 0.05	1.95 ± 0.04
Zn2-H250	4.26 ± 0.35	4.28 ± 0.31	4.32 ± 0.32	5.19 ± 2.92	4.40 ± 0.30

**Table 5:** Average distances (Å) between Zn and their coordinating atoms, collected from the trajectory of NDM-1 and L218T mutant complexes.

	NDM-1/Meropenem Complex	L218T/Meropenem Complex
Zn1-Zn2	4.54 ± 0.15	5.87 ± 0.42
Zn1-H120	3.63 ± 0.56	4.04 ± 0.78
Zn1-H122	1.95 ± 0.06	4.34 ± 0.98
Zn1-H189	2.05 ± 0.10	1.92 ± 0.07
Zn2-D124	1.73 ± 0.04	1.84 ± 0.33
Zn2-C208	1.93 ± 0.03	1.96 ± 0.04
Zn2-H250	1.97 ± 0.07	2.36 ± 0.31

## Data Availability

Not applicable.

## Supplementary Materials

The following are available online, Table S1: Primers used in this study, Figure S1: Fluorescence spectra, Figure S2: Root Mean Square Deviation (RMSD) of backbone atoms of the investigated free proteins and Figure S3: Root Mean Square Deviation (RMSD) of backbone of wt complex and L218T mutant complex.

## Sample Availability

Samples of the compounds are available from the authors.
