# Supplementary material for: Exploring the Role of L10 Loop in New Delhi Metallo-β-lactamase (NDM-1): Kinetic and Dynamic Studies
Source: Molecules. 2021 Sep 9;26(18):5489. doi: 10.3390/molecules26185489 (PMC8467308; doi:10.3390/molecules26185489)

Table 1S: Primers used in this study

Primers	5’ nucleotide sequence	T_m
L218T_for	gccaagtcgaccggcaat	58°C
L218T_rev	attgccggtcgacttggc	58°C
L221T_for	ggcaataccggtgatgcc	58°C
L221T_rev	ggcatcaccggtattgcc	58°C
L269H_for	atggccgacaagcatcgctga	60°C
L269H_rev	tcagcgatgcttgtcggccat	60°C
Y229W_for	actgagcactgggccgcgtca	62°C
Y229W_rev	tgacgcggcccagtgctcagt	62°C
NDM_for	ggggg**catatg**ggtgaaatccgcccga	58°C
NDM_rev	ggggg**ctcgag**tcagcgcagcttgtcggc	58°C

Restriction site sequence is in bold.

Figure 1S: Fluorescence spectra

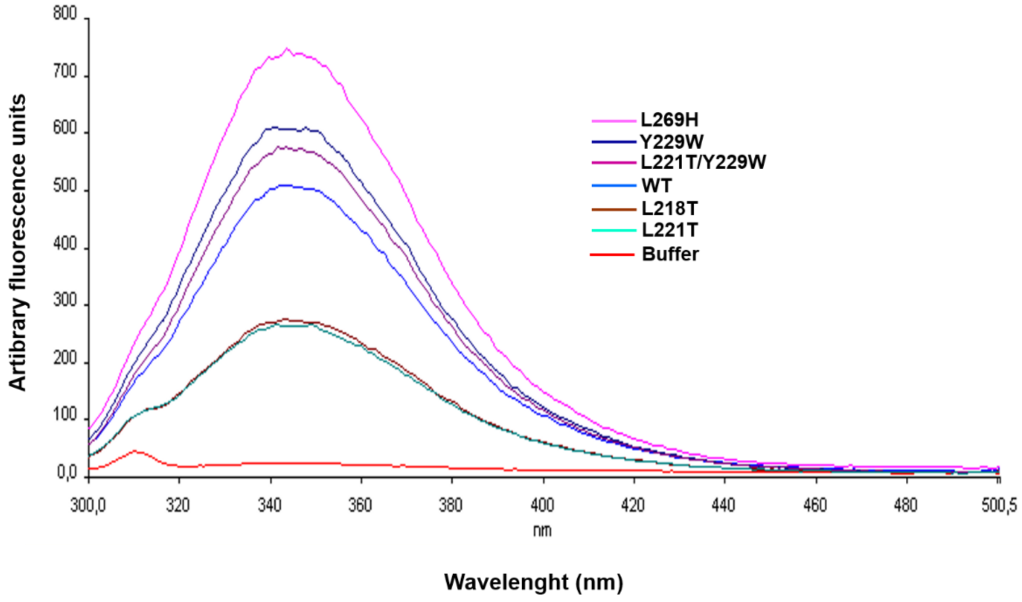

Figure 2S: Root Mean Square Deviation (RMSD) of backbone atoms of the investigated free proteins.

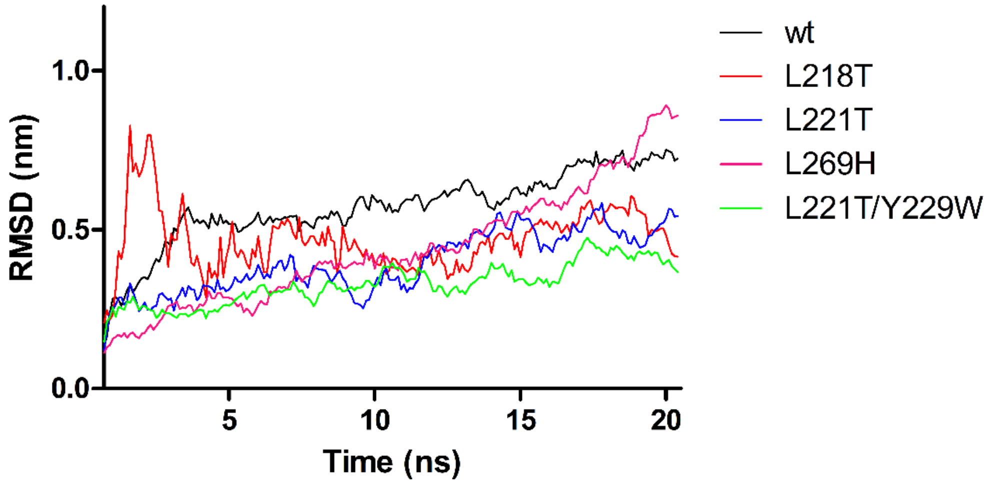

Figure 3S: Root Mean Square Deviation (RMSD) of backbone of wt complex and L218T mutant complex.

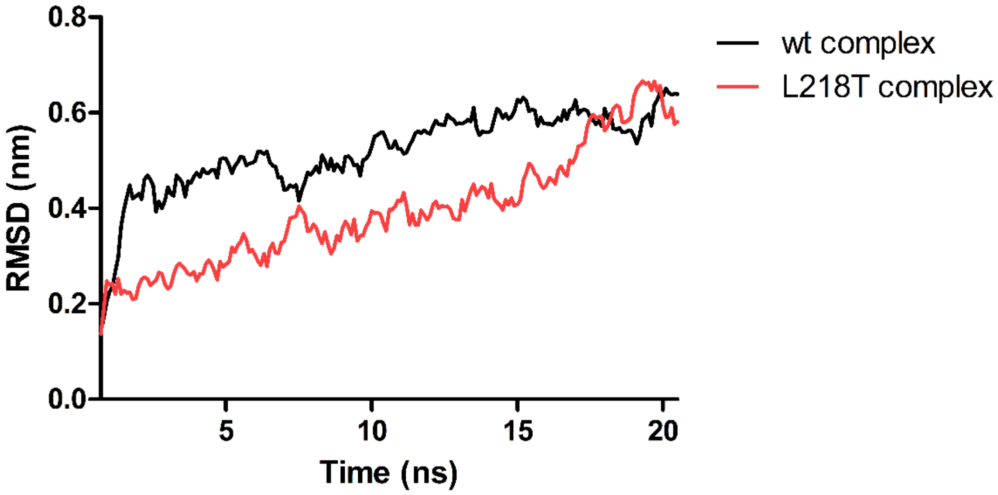

Supplement: Supplementary file 1 [file molecules-26-05489-s001.zip › molecules-1327849-supplementary.pdf]
